# Toshisada Nishida (1941–2011): Chimpanzee Rapport

**DOI:** 10.1371/journal.pbio.1001185

**Published:** 2011-10-25

**Authors:** Frans B. M. de Waal

**Affiliations:** Living Links, Yerkes National Primate Research Center, Emory University, Atlanta, Georgia, United States of America

## Abstract

Frans de Waal pays tribute to pioneering primatologist Toshisada Nishida, who transformed our understanding of chimpanzee behavior and culture and galvanized efforts to ensure their conservation.

*“Chimpanzees are always new to me.”*

Toshisada Nishida [1]

One of the absolute greats of primatology, Toshisada Nishida (March 3, 1941–June 7, 2011), recently passed away at the age of 70 (Figure 1). We have come such a long way in our knowledge of chimpanzees, and the discoveries have reached us in such a gradual and cumulative fashion, that it is easy to forget how little was known when Nishida set out for Africa to establish one of the first chimpanzee field sites, in 1965. At the time, chimpanzees did not yet occupy the special place in our thinking about human evolution reserved for them today. Science considered baboons the best model of human evolution, since baboons had descended from the trees to become savanna-dwellers, like our ancestors. These rambunctious monkeys, however, are genetically more distant from us, and many of the characteristics deemed important for human evolution are either absent or minimally developed, such as tool technology, cooperative hunting, food sharing, territoriality, cultural traditions, and certain cognitive capacities, such as planning and theory-of-mind. Chimpanzees show all of them.

**Figure 1:**
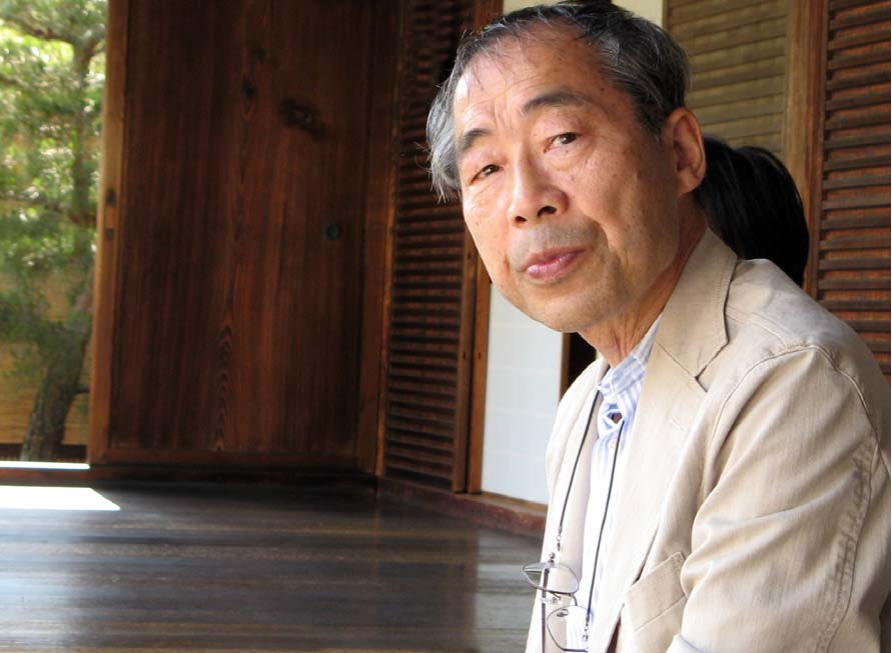
Toshisada Nishida in a Kyoto temple, in 2007. Photograph by Frans de Waal.

Early primatologists had seen chimpanzees travel through the trees, eating fruits at their leisure, but rarely noticed anything of interest in their behavior. This was partly due to low visibility and the apes' wariness of people. The study of chimpanzee behavior in nature began in earnest only in the 1960s with one field site set up by Jane Goodall in Gombe Stream, and another one 120 km to the south by Japanese scientists led by Nishida. Both teams were in it for the long haul rather than the brief expeditions others had previously undertaken.

Nishida started his career inspired by the legendary father of Japanese primatology, Kinji Imanishi, at Kyoto University. As a graduate student of Imanishi's successor, Junichiro Itani, Nishida first studied Japanese macaques before he traveled to Tanzania. He chose a forest in the foothills of the Mahale Mountains, at Lake Tanganyika, where he waited patiently for chimpanzees at a patch of sugar cane planted to attract them. The primates started to make regular visits only after about six months. Based on his field observations, Nishida defended his dissertation at Kyoto University in 1968. He occupied a teaching position at Tokyo University, from 1969 until 1988, before he became Full Professor in the Zoology Department of Kyoto University from 1988 until 2004. After his retirement from this position, he became Director of the Japan Monkey Center.

One of Nishida's first discoveries was truly groundbreaking. While science still described the chimpanzee as a peaceful vegetarian that roamed the forest without any need for social bonds—not unlike Rousseau's noble savages—Nishida had noticed that chimpanzees live a communal life with territorial boundaries and perhaps even hostility between neighboring unit-groups. This was not an easy discovery, because chimpanzees are often encountered alone or in small groups in the forest. One can determine community relations only if one recognizes all individuals and keeps careful track of their travels. Nishida's discovery upset not only Western notions of chimpanzees as individualists, but also the expectations of his Japanese mentors who thought chimpanzees would live, like humans, in nuclear family–like arrangements. Debate about what to expect must have been heated, because when Nishida's teacher Itani arrived in Kigoma, the student couldn't wait for their physical reunion to shout from aboard the steamship Liemba: “There is no familoid in the chimpanzee society.” Itani-sensei shouted back, “That can't be true!”

We have learned much about chimpanzees since then, such as that they hunt and eat meat, that they raid neighboring territories, that they have complex tool skills that differ from group to group, that they medicate themselves with plants, that males engage in power politics while competing over status and females, and so on. The list of discoveries is impressive and the Mahale field site has been central in furnishing the evidence. From the start, the approach followed at Mahale has been that of Imanishi, who urged his students to identify individuals, give them names, and follow them over time. Not just for weeks or months, as done previously, but for years and years, so that one could understand the kinship relations within the community. With a species that breeds as slowly and is as long-lived as the chimpanzee, one needs to follow individuals for a long time indeed to know whether or not two adult males are brothers or how many offspring a female rears during her lifetime. Before scientists learned to analyze DNA from fecal or hair samples, the only way to determine kinship relations was to hunker down for the long term.

At the time, habituation of chimpanzees was typically done by means of food provisioning. Nishida first tried sugarcane, until he found that bananas worked better. He developed a “mobile provisioning” technique, in which scientists announced their presence to distant chimpanzees by imitating the species-typical hooting calls, after which the apes would approach and obtain food. This way, their normal roaming patterns remained intact, as they never attached themselves to a fixed feeding site. After the feeding session, the investigators followed the apes for the rest of the day.

Some scientists have criticized food provisioning as a technique that makes chimpanzees more aggressive, using this as an argument against reports of lethal intergroup warfare. In the 1970s, these reports engendered fierce debate about the aggressive nature of our own species. If chimpanzees kill each other like we humans do, so the argument went, we probably inherited our territorial tendencies from the ancestor shared with chimpanzees. Opponents of this view blamed the violence of wild chimpanzees on food provisioning. But while provisioning in Mahale ended in 1982, aggressive behavior of the chimpanzees towards neighbors hardly changed. Moreover, violent behavior has also been reported from field sites where researchers *never* provisioned chimpanzees. For this reason, there is little doubt among experts that chimpanzee males are naturally violent [2].

A true pioneer of chimpanzee field research, Nishida inspired many students, collaborated with numerous international colleagues, and left one of the most impressive publication records of any primatologist (a complete list can be found in [3]). Nishida was a most dedicated scientist who in his early career spent years, and later many months per year, under relatively primitive circumstances, without tap water or electricity, at Kasoje, at the foot of the mountains. As a result, he knew all chimpanzees in several groups, and by “knowing”, I mean that he observed them as infants, saw them grow up as juveniles, and followed them through their prime into old age.

In 1982, at the same time that I wrote *Chimpanzee Politics* [4], Nishida and his students were documenting very similar power struggles among wild chimpanzees [5],[6], including one by a male named Kalunde. Kalunde played a game that Nishida called “allegiance-fickleness,” which allows over-the-hill males to carve out a key position by regularly switching sides in alliances with younger adult males. It was a thrill for me to meet Kalunde on a visit to the Mahale Mountains, and see Nishida in action. Nishida lived up to his reputation in the field, being incredibly knowledgeable not only about the primates he studied, but about the forest as a whole and all of the flora and fauna therein. Not satisfied with bookish knowledge, he personally tasted each and every new leaf or fruit that he saw his chimpanzees consume—the ultimate act of identification with one's subjects.

Nishida had met “my” chimpanzees about a decade before I met “his.” When he visited the Yerkes National Primate Research Center's Field Station, in Georgia, I showed him around the way I do many guests. One big difference, however, was the reaction of the chimpanzees. Normally, they do not like strangers, which they express by spitting, throwing, displaying, and the like. But with Nishida, there was no reaction at all. He was standing next to me, leaning sideways a little, walking quietly without abrupt moves as he also did at Mahale, and the apes seemed to think that this man was perfectly all right.

One major advance in the study of chimpanzee habits came when Nishida discovered that wild chimpanzees consume *Aspilia* leaves. These leaves have no known nutritional value, and are in fact not digested. The chimpanzees consume them very slowly, mostly in the morning, swallowing the leaves without chewing. Together with Richard Wrangham, the first Western primatologist to set foot in Mahale, in 1971, Nishida published his observations of potentially medicinal use of plants by wild chimpanzees, thus founding the new field of *zoopharmacognosy* (i.e., self-medication by animals ingesting plants, insects, or soils) [7].

Another important moment in primatology occurred when William McGrew and Caroline Tutin visited Mahale in 1975. Extensively familiar with the chimpanzees in Gombe National Park, they had no reason to expect striking behavioral differences in the same subspecies with the same ecology. Nevertheless, the Mahale chimpanzees frequently engaged in hand-clasp grooming, whereas this behavior is entirely unknown from Gombe. One chimpanzee takes the hand of another, which they then lift above their heads, while both groom each other's armpits with their free hands. Based on their visit, McGrew and Tutin were the first to seriously question the assumption of “typical” chimpanzee behavior, an important step towards culture studies on the great apes [8].

Nishida fostered many such contacts, and was central in bringing Japanese primatology and Western scientists together. He stimulated his students and colleagues to write in English, and was himself the first Japanese primatologist to publish an entire article in this language. He was first author or editor on no less than 17 books and volumes, mostly in Japanese, but the last two in English. His work was characterized by great attention to the smallest details, resulting in comprehensive catalogues of behavior patterns [9]. Nishida's magnum opus, *Chimpanzees of the Lakeshore: Natural History and Culture at Mahale*, will soon be released by Cambridge University Press.

Nishida was one of the world's most respected primatologists, and was presented in 2008, along with Jane Goodall, with the prestigious Leakey Prize of the L. S. B. Leakey Foundation, which recognizes accomplishments in human evolutionary science. He was President of the Primate Society of Japan, President of the International Primatological Society, and Editor-in-Chief of the journal *Primates*.

In March 2004, I attended Nishida's retirement reception at Kyoto University. His lecture for a full room was riveting, especially given the historical details of how our knowledge has grown over the years and the critical role Japanese scientists have played in modern primatology [10]. Many friends and colleagues from all over the world had come, because Nishida fostered excellent relations and was active in the conservation movement. He successfully lobbied the Tanzanian government to accord the Mahale Mountains the status of National Park, in 1985, and established the Mahale Wildlife Conservation Society, in 1994. He also led the attempt with the UNESCO to make the great apes a “World Heritage Species.” His face always lit up when he talked of chimpanzees, so there was no doubt how close to his heart they were.

He remained dedicated to primatology and the protection of primates to the very end. He made his last field trip to Mahale in the summer of 2009. A few months before his death of cancer, which he had foreseen for several years, he called in two of his trusted students, now professors themselves, telling them that all he wanted from them was to make sure the Mahale project would continue for at least another century [11].
